# Intracranial Bleeding in a Female Hemophilia Patient: Molecular Analysis of the *Factor 8* Gene and Determination of a Novel Mutation

**DOI:** 10.4274/tjh.2017.0385

**Published:** 2018-08-05

**Authors:** Burçak Tatlı Güneş, Zühal Önder Siviş, Eda Ataseven, Barış Malbora, Meral Türker, Fatma Burcu Belen, Berna Atabay, Tahir Atik, Esra Işık, Ferda Özkınay

**Affiliations:** 1 İzmir Tepecik Training and Research Hospital, Clinic of Pediatric Hematology, İzmir, Turkey; 2 Başkent University Faculty of Medicine, Department of Pediatric Hematology, Ankara, Turkey; 3 Ege University Faculty of Medicine, Department of Pediatric Genetics, İzmir, Turkey

**Keywords:** Hemophilia, Intracranial bleeding, Female

## To the Editor,

**Figure 1:**
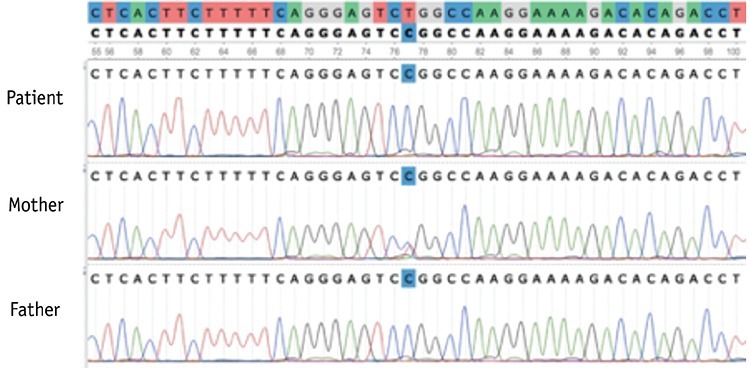
Sanger sequencing confirmation: (a) the mother (b), the father, and (c) the patient show heterozygous, hemizygous, and homozygous c.608T>C (L203P) mutation in the *F8* gene.

An 11-month-old female patient was admitted to the emergency department with right occipital fracture and epidural hematoma. The father had severe hemophilia A and the parents were cousins. Laboratory tests revealed normal complete blood count and prolonged activated partial thromboplastin time. Mixing test results were normalized after mixing with normal plasma. After plasma samples were collected for further diagnostic tests, fresh frozen plasma and dexamethasone were administered. The factor VIII level was 0.1%, 35%, and 0.5% for the patient, mother, and father, respectively. The patient’s von Willebrand factor (VWF) level was 128 IU/mL, VWF:Ricof was 110 IU/mL, collagen ADP was 110 (reference: 71-118) s, and collagen epinephrine was 98 (reference: 85-165) s. Intron 22 inversion was investigated with the IS-PCR method and was found to be normal. Whole-genome analysis including all exonic regions of the *F8* gene (NM_000132.3) was conducted and the homozygous c.608T>C (L203P) mutation was found. This mutation was not previously reported. As this variant was not reported in any exome databases (ExAC, EVS) and as it was shown to be the cause of the disease in at least three in silico protein modeling programs, the mutation was considered as a novel mutation causing hemophilia A (“probably damaging” with 0.987 PolyPhen2 score, “disease causing” with 0.999 MutationTaster score, and “damaging” with 0 SIFT score). The mutation was also confirmed by Sanger sequencing (Figure 1). Plasma-derived FVIII at 2x500 IU/day was administered for 14 days followed by 300 IU/week prophylaxis. Inhibitor screening at the 5^th^ and 10^th^ exposure days was negative.

**Figure 2:**
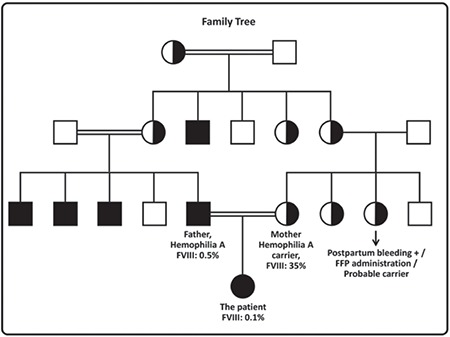
Family tree. The patient was homozygous, the father hemizygous, and mother heterozygous for the c.608T>C (L203P) mutation.

Hemophilia A is rarely seen in female patients due to skewed inactivation of the X chromosome leading to inactivation of the wild-type X chromosome, anomalies like Turner syndrome, or translocations, as well as homozygous/compound heterozygous mutations for hemophilia A [1,2,3,4,5]. The karyotype analysis of our patient revealed 46,XX. The patient and the father were hemizygous and mother was heterozygous for the c.608T>C (L203P) mutation (Figure 2). The clinical situation of our patient as she was admitted with epidural hematoma requiring surgical intervention and the fact that the family did not apply for prenatal diagnosis before birth point out the importance of prenatal diagnosis in regions where consanguineous marriage is common.
